# Supplementary material for: Synthesis of Celecoxib-Eutectic Mixture Particles via Supercritical CO2 Process and Celecoxib Immediate Release Tablet Formulation by Quality by Design Approach
Source: Pharmaceutics. 2022 Jul 26;14(8):1549. doi: 10.3390/pharmaceutics14081549 (PMC9331714; doi:10.3390/pharmaceutics14081549)
Supplement: Supplementary file 1 [file pharmaceutics-14-01549-s001.zip › pharmaceutics-1771043-supplementary.pdf]

Supplementary Materials:

Synthesis of Celecoxib-Eutectic Mixture Particles via Supercritical CO_2 Process and Celecoxib Immediate Release Tablet Formulation by Quality by Design Approach

Seung-Hyeon Hong [1,2,†], Linh Dinh [1,2,†], Sharif Md Abuzar [1,2], Eun Seok Lee [1,2] and Sung-Joo Hwang [1,2,*]

[1] College of Pharmacy, Yonsei University, 85 Songdogwahak-ro, Yeonsu-gu, Incheon 21983, Korea; shhongmartin@naver.com (S.-H.H.); dinhkhanhlinh@yonsei.ac.kr (L.D.); sumonzar@gmail.com (S.M.A.); leslech@naver.com (E.S.L.)

[2] Yonsei Institute of Pharmaceutical Sciences, Yonsei University, 85 Songdogwahak-ro, Yeonsu-gu, Incheon 21983, Korea

* Correspondence: sjh11@yonsei.ac.kr; Tel.: +82-32-7494518

† These authors contributed equally to this work.

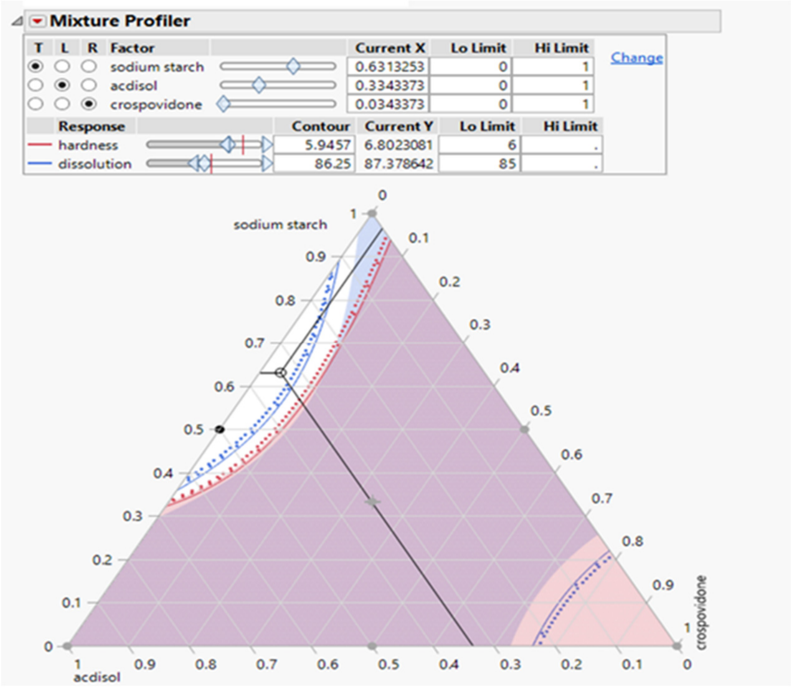

Figure S1. DoE result of the ratio of sodium starch glycolate, cross-linked carboxyl methyl cellulose sodium (Ac-Di-Sol®) and cross-linked povidone in CEL-ADI EM IR tablet analyzed by JMP® software.

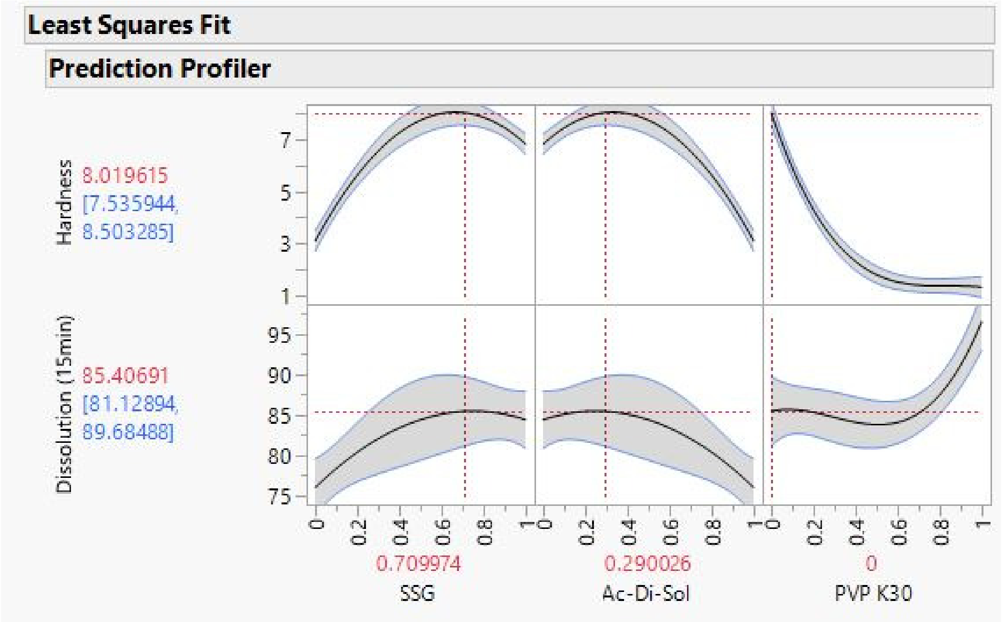

Figure S2. DoE result of the ratio of sodium starch glycolate, cross-linked carboxyl methyl cellulose sodium (Ac-Di-Sol®) and cross-linked povidone in CEL-ADI EM IR tablet, responses predicted by JMP® software.
